# Local Indicators of Spatial Autocorrelation (LISA): Application to Blind Noise-Based Perceptual Quality Metric Index for Magnetic Resonance Images

**DOI:** 10.3390/jimaging5010020

**Published:** 2019-01-15

**Authors:** Michael Osadebey, Marius Pedersen, Douglas Arnold, Katrina Wendel-Mitoraj

**Affiliations:** 1 Department of Computer Science, Norwegian University of Science and Technology, Teknologivegen 22, N-2815 Gjøvik, Norway; 2 Montreal Neurological Institute and Hospital, McGill University, 3801 University St, Montreal, QC H3A 2B4, Canada; 3 BrainCare Oy, Finn-Medi 1, PL 2000, 33521 Tampere, Finland

**Keywords:** magnetic resonance imaging (MRI), image quality, noise, local indicators of spatial autocorrelation (LISA), local moran statistics (LMS), global moran statistics (GMS), perceptual quality, contrast, sharpness

## Abstract

Noise-based quality evaluation of MRI images is highly desired in noise-dominant environments. Current noise-based MRI quality evaluation methods have drawbacks which limit their effective performance. Traditional full-reference methods such as SNR and most of the model-based techniques cannot provide perceptual quality metrics required for accurate diagnosis, treatment and monitoring of diseases. Although techniques based on the Moran coefficients are perceptual quality metrics, they are full-reference methods and will be ineffective in applications where the reference image is not available. Furthermore, the predicted quality scores are difficult to interpret because their quality indices are not standardized. In this paper, we propose a new no-reference perceptual quality evaluation method for grayscale images such as MRI images. Our approach is formulated to mimic how humans perceive an image. It transforms noise level into a standardized perceptual quality score. Global Moran statistics is combined with local indicators of spatial autocorrelation in the form of local Moran statistics. Quality score is predicted from perceptually weighted combination of clustered and random pixels. Performance evaluation, comparative performance evaluation and validation by human observers, shows that the proposed method will be a useful tool in the evaluation of retrospectively acquired MRI images and the evaluation of noise reduction algorithms.

## 1. Introduction

Noise is a type of distortion which is observed as the random variation of pixel intensity levels within an image. Its presence is undesirable because it can obscure useful information and degrade perceived visual quality required for the diagnosis, treatment and monitoring of diseases [1]. Sources of noise in magnetic resonance imaging (MRI) system images can be broadly classified into physiological noise and electronic noise [2,3]. Physiological noise arise from the imaged subject while electronic noise originates from system components such as the radio frequency (RF) coils.

There are several reasons to justify the need for noise-based quality evaluation methods. Image quality evaluation is a nontrivial task. Since noise is one of the several types of distortion that can degrade an image, noise-based quality evaluation method can be efficiently combined with other distortion-specific quality evaluation methods to evaluate the quality of an image. Some operating conditions, applications and acquisition techniques encourage noise to dominate over other types of distortion. During image acquisition for the elderly or patients in trauma, noise can dominate because concern for patient comfort requires trade-off between length of scan time and image resolution. Parallel imaging (PI) acquisition technique is vulnerable to noise because its primary purpose is to reduce acquisition time through acquiring and averaging fewer data points [4]. In image processing application, the regularization parameters incorporated in most denoising algorithms, to allow a balance between noisiness and blurriness, can be easily optimized using noise-based quality evaluation methods.

Current contributions on noise-based MRI quality evaluation can be classified as traditional, model-based, Moran statistics and window-based Moran coefficient methods. The traditional methods include signal-to-noise ratio (SNR), peak signal-to-noise ratio (PSNR), mean square error (MSE) and root mean square error (RMSE). Model-based methods assess the quality of a noisy MRI image by estimating the noise variance. Model-based methods include the report in [5] which adapt the median absolute deviation (MAD), originally proposed to estimate Gaussian noise in the wavelet domain, to estimate noise in MRI images. In [6], the level of noise was estimated from the distribution of local moments. The approach in [7] estimate noise from a Rician distribution. Knowledge of the Rician distribution is obtained by double acquisition of same image. The statistics of the background signal in MRI images is exploited in [8] for the estimation of noise. The report in [9] estimates noise level from a mathematical model that describe the relationship between the clique potential of Markov random fields and noise variance. The report in [10] uses maximum likelihood principle to estimate noise in local regions of the image. Moran statistics method include the contribution by [11] which evaluates a noisy image by performing joint-count statistics and Moran test on a residual image. The residual image is formed by subtracting the original image from its Gaussian smoothed version. Window-based Moran coefficient methods slide a fixed-size window through an image. Thereafter, Moran coefficients computed from local regions are combined to measure the sharpness quality of the image. Techniques in this category include the contributions by [12,13,14,15]. The report in [16] applied machine learning technique to perceptual quality assessment of MRI images. It modifies the popular Blind/Referenceless Image Spatial Quality Evaluator (BRISQUE) [17] by training regression model of MRI image features and subjective mean opinion scores.

The appropriateness of traditional full-reference methods as indicators of average error has raised concerns among researchers [18,19,20,21]. The concerns arose from multiple definitions associated with each metric, resulting in different interpretations. Multiple definitions of a quality index makes it difficult to compare results from different imaging systems, modalities and researchers [21]. Furthermore, the traditional methods are not formulated from structural and spatial information, an important requirement for perceptual image quality. Traditional methods and window-based Moran coefficient methods operate only in applications where a reference image is available. No-reference technique is the most practical and realistic approach to quality evaluation because in the real-world there is no perfect image [22]. Model-based techniques evaluate image quality by estimating noise variance. Noise variance, a physical attribute of an image, does not directly translate to perceptual image quality. Model-based quality methods such as [8] will be unreliable where there is a limited or corrupted background signal. In most current techniques, noise in the foreground is assumed to follow Gaussian and Rician distributions at high and low SNR, respectively. Since there is no clearly defined threshold which demarcates low and high SNR, this assumption can introduce modeling error which compromises accurate estimation of noise. Additional resources such as pre-correction [23] and post-correction [24] techniques are required to retrieve correct noise estimates.

Noise is one of many attributes which combine to give an image its perceptual quality. Even in noise-dominant environment, it is very difficult to estimate noise. Since noise contributes to visual perception, we are of the opinion that it is more practical to evaluate the perceptual quality of a noisy image. In this paper, we propose to combine global Moran statistics and local indicators of spatial autocorrelation (LISA) [25] for a no-reference perceptual quality evaluation of noisy grayscale images such as MRI images. LISA, introduced by Luc Anseli in 1995 to the geographical analysis community, is an extension of Moran statistics [26] developed by Patrick Moran in 1948. LISA is applied for the identification of local patterns of spatial association in mapped data and for the decomposition of global measures of autocorrelation such as Moran’s statistics [26], Geary’s statistics [27] and Getis-Ord G statistics [28]. LISA can be regarded as a variant of window-based Moran coefficient proposed by [11] for pixels within a local region in an image. Furthermore, LISA extracts structural information and provide spatial variation of image quality, thus. satisfying the requirement of a good perceptual quality indicator outlined in [12].

This paper is organized as follows. Section 2 and Section 3 describe our method and experiment on performance evaluation for the proposed quality assessment. Performance evaluation results are displayed in Section 4. Results from the experiment are discussed in Section 5. Section 6 concludes this report.

## 2. Methods

### 2.1. Problem Formulation

#### 2.1.1. Noise, Sharpness and Contrast in Grayscale Images

Clustered pixels in grayscale images allow human observers and man-made devices to distinguish between the different anatomical structures and also distinguish between normal and abnormal structures under different pathological conditions. The degree of clustering measures the level of contrast and sharpness quality attributes of an image. Contrast is the perceived visual differences between the different structures within the image [29]. Sharpness is the visibility of small structures as measured by the deviation and difference of grey levels in the image [30].

Noise influences the contrast and sharpness quality attributes of medical images [31]. Rician noise in grayscale images such as MRI images induces randomness which causes break-up of clustered pixels and erode edges. Erosion of edges reduces contrast between different structures thereby making it difficult to utilize images for disease diagnosis. A global measure of spatial autocorrelation cannot provide information on the variations in locally perceived visibility of noise because it assumes uniform cluster patterns throughout the image. Therefore, quantification of local spatial autocorrelation will be a useful parameter for noise-based quality evaluation.

#### 2.1.2. Local Indicators of Spatial Autocorrelation (LISA) Statistics

The most popular LISA statistics are the local Moran statistics (LMS). Henceforth LISA statistics will be used interchangeably with LMS throughout this paper. The feature of interest is pixel intensity level $x_{i}$ at locations i∈{1,2,⋯,N} in an image. Each pixel location *i* is the center of a neighbourhood defined by a fixed-size r×c window. In each neighbourhood, $x_{j}$ are the neighbouring pixels to $x_{i}$. The LMS $I_{i}$ in each neighbourhood is expressed as:

(1) $$I_{i}=z_{i}\sum_{j}w_{ij}z_{j}$$

where $z_{i}$, $z_{j}$ are the deviations of $x_{i}$, $x_{j}$, respectively from the mean pixel intensity level $\bar{X}$ within the neighbourhood:

(2) $$\begin{array}{c} z_{i}=\frac{\left(x_{i}-\bar{X}\right)}{S_{i}^{2}}\\ z_{j}=\left(x_{j}-\bar{X}\right) \end{array}$$

where $w_{ij}$ is a spatial weight matrix which define the spatial interaction between $x_{i}$ and its neighbouring pixels $x_{j}$, and $S_{i}^{2}$ is the standard deviation of pixels $x_{j}$ that are neighbours to $x_{i}$:

(3) $$S_{i}^{2}=\sum_{j=1}^{N}\frac{{\left(x_{j}-\bar{X}\right)}^{2}}{N}$$

The followings characterize the information that can be derived from LISA statistics:

1. Average of $I_{i}$ across the image gives the global Moran Statistics (GMS) *I*.
  (4) $$I=\frac{1}{N}\sum_{i}I_{i}$$
  The range of values for Global Moran statistics is {+1≤I≤−1}. For gray level images, GMS of 1 indicates the highest degree of clustering, GMS of 0 indicates randomness and GMS of −1 indicates highest degree of randomness and dispersion:
  (5) $$I=\left\{\begin{array}{ll} 1 & \mathrm{Highest}\;\mathrm{Degree}\;\mathrm{of}\;\mathrm{Clustering}\\ 0 & \mathrm{Randomness}\\ -1 & \mathrm{Highest}\;\mathrm{Degree}\;\mathrm{of}\;\mathrm{Randomness}\;\mathrm{and}\;\mathrm{Dispersion} \end{array}\right.$$
  The range of values for LMS is $\left\{+k_{1}\leq I\leq -k_{2}\right\}$, where $k_{1}$, $k_{2}$, the upper and lower limits of LMS is determined by the type of grayscale image and the distribution of pixel intensity levels.
2. LISA statistics can identify the presence of outliers and the degree of spatial clustering at specific location.
3. The magnitude of positive and negative values of LISA statistics measures the degree of pixel clusters and pixel dispersion, respectively, at specific location.
4. Positive values of LISA statistics are an indication of clustering; there is relatively low margin between the pixel intensity level at specified location and corresponding intensity levels of neighbouring pixels. On the other hand negative values of LISA statistics indicates the presence of an outlier; there is a relatively wide margin between the intensity of pixel at specified location and corresponding intensity levels of neighbouring pixels.
5. Clustered pixels can be classified as clusters of high pixel intensity levels HH and clusters of low pixel intensity levels LL. Outliers can be classified as pixels with high intensity values surrounded primarily by pixels of low intensity values HL and pixels of low intensity values surrounded primarily by pixels of high intensity values LH.

### 2.2. Implementation

**Figure 1:**
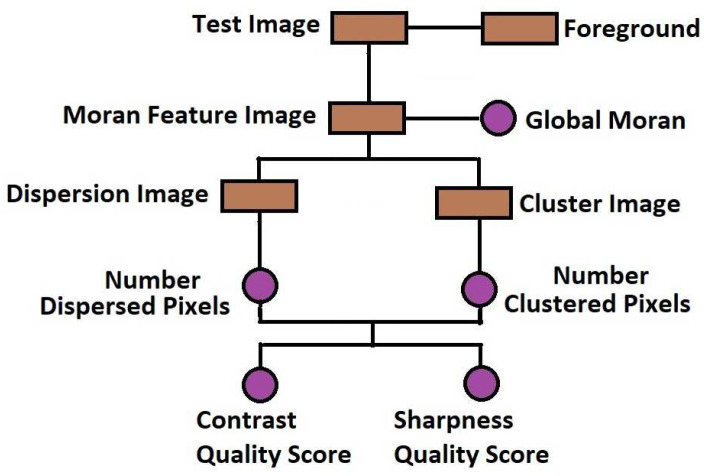
Flow chart of the proposed method for blind noise-based quality assessment in MRI images.

**Figure 2:**
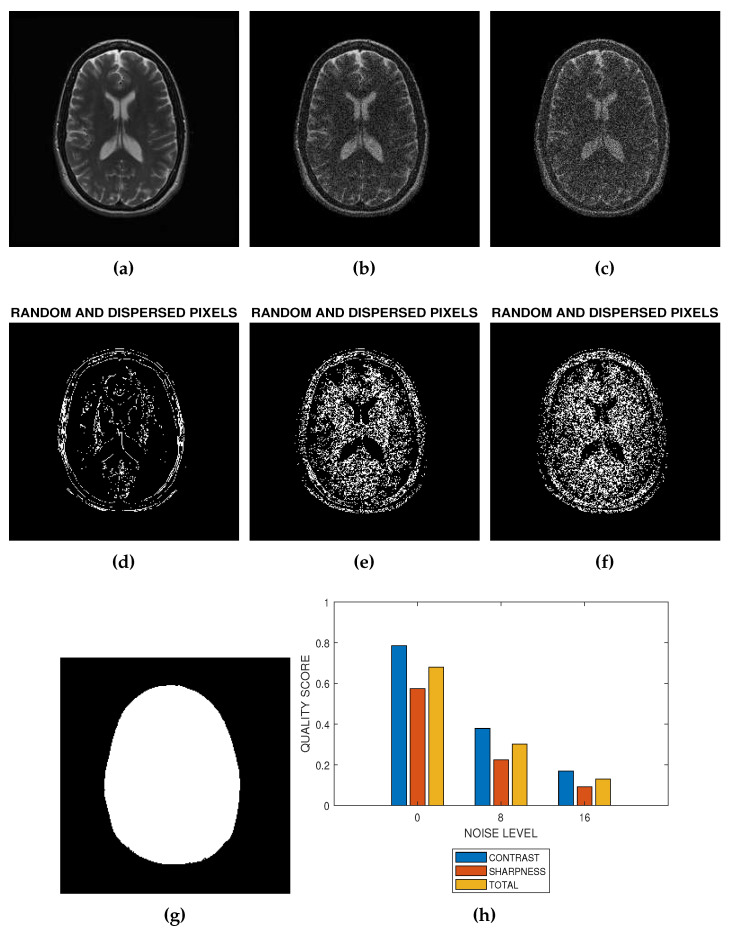
The implementation of blind noise-based quality assessment in MRI images. (**a**) The test image provided by NeuroRx Research Inc. (**b**) Degraded version of the test image in (**a**) at 8 percent Rician noise. (**c**) Degraded version of the test image in (**a**) at 16 percent Rician noise. (**d**) Random and dispersed pixels in the test image in the absence of noise distortion. (**e**) Random and dispersed pixels in the test image at 8 percent Rician noise. (**f**) Random and dispersed pixels in the test image at 16 percent Rician noise. (**g**) The Foreground extracted from the test image in (**a**). (**h**) Variation of contrast, sharpness and total quality scores at Rician noise levels of 0 percent, 8 percent and 16 percent.

The flowchart in Figure 1 and the images in Figure 2 describe how the proposed method can be implemented. Figure 2 is a slice from a T2 MRI volume data provided by NeuroRx Research Inc. (Montreal, QC, Canada). The four successive steps to implement the algorithm are outlined below.

1. Step 1: Foreground Extraction
  Foreground extraction is the extraction of the regions of interest in the test image from the background region. Foreground image $I_{f}$ was extracted using the threshold method. There are three steps to extract the foreground. First is global threshold. The threshold was set at the mean intensity level of the image. The next step is a morphological filling operation followed by area threshold where small regions within the image are eliminated. Knowledge of the foreground shown in Figure 2g allows the determination of the indices of pixels as well as the total number of spatial locations in the foreground region. The number of spatial locations is required in the later implementation steps.
2. Step 2: Feature Extraction
  The local Moran feature image is derived by computing the local Moran statistics of the test image according to Equation (1). The spatial weight $w_{ij}$ which define the interaction of pixels is determined by the kernel dimension. In this research, the spatial weight was implemented using a 3×3 kernel. The local Moran statistics is averaged according to Equation (4) to obtain the global Moran statistics.
3. Step 3: Feature Classification
  Using global threshold, the local Moran feature image $I_{LMF}$ is classified into two classes. The first class consist of random and dispersed pixels $I_{LMF_{A}}$. The second class $I_{LMF_{B}}$ consist of clustered pixels:
  (6) $$I_{LMF}=\left\{\begin{array}{ll} I_{LMF_{A}} & \mathrm{if}\;I_{i}\leq 0\;\\ I_{LMF_{B}} & \mathrm{otherwise} \end{array}\right.$$
  The two classes of pixels are calculated over the foreground region. Figure 2a,b are the images resulting from the addition of 8 percent and 16 percent Rician noise levels to the image in Figure 2a. Random and dispersed feature images corresponding to noise level of 0 percent (Figure 2a), 8 percent (Figure 2b) and 16 percent (Figure 2c) are displayed in Figure 2d–f, respectively.
4. Step 4: Quality Prediction
  Quality prediction is based on two concepts. First, the GMS is considered a perceptual weight which modulates the LMS. Second, the test image is a real grayscale image having heterogeneous features, that is, images in which pixels can be assigned to at least two different classes. In contrast, sharpness and total quality scores shown in Figure 2h are predicted from the perceptually weighted sum of the clustered and dispersed pixels within a grayscale image.
  The contrast quality score $Q_{1}$ is defined as:
  (7) $$Q_{1}=I\left(1-\frac{N_{C_{A}}}{N_{I_{f}}}\right)+\left(1-I\right)\left(\frac{N_{C_{B}}}{N_{I_{f}}}\right)$$
  where $N_{C_{A}}$, $N_{C_{B}}$, $N_{I_{f}}$ are the number of dispersed, clustered and foreground pixels, respectively in the image.
  The sharpness quality score $Q_{2}$ is defined as:
  (8) $$Q_{2}=1-\left(\frac{N_{C_{A}}}{N_{I_{f}}}\right)+\left(1-I\right)\left(\frac{N_{C_{B}}}{N_{I_{f}}}\right)$$
  The total quality score $Q_{T}$ is the average of the contrast $Q_{1}$ and sharpness $Q_{2}$ quality scores:
  (9) $$Q_{T}=\frac{\left(Q_{1}+Q_{2}\right)}{2}$$
  Here, we show how the quality scores defined in Equations (7) and (8) can predict the contrast and sharpness quality scores of an ideal, extremely degraded and real MRI slices.
  (a) Ideal MRI Slice
    In an ideal MRI slice, the pixels tend towards the highest degree of clustering. According to Equation (5),
    (10) I≈1
    Since random and dispersed pixels are sparse in an ideal MRI image,
    (11) $$N_{C_{A}}\approx 0$$
    Inserting Equations (10) and (11) into Equations (7) and (8), the contrast and sharpness quality scores are both equal and optimized towards a value of 1,
    (12) $$Q_{1}=Q_{2}\approx 1$$
  (b) Extremely Degraded MRI Slice
    For an extremely degraded MRI slice, the pixels tend towards the highest degree of randomness and dispersion. According to Equation (5),
    (13) I≈−1
    Random and dispersed pixels are dominant and contained within the foreground region,
    (14) $$N_{C_{A}}\approx N_{I_{f}}$$
    Clustered pixels are sparse, thus
    (15) $$N_{C_{B}}\approx 0$$
    Inserting Equations (13)–(15) into Equations (7) and (8), the contrast and sharpness quality scores are both equal and minimized towards a value of 0,
    (16) $$Q_{1}=Q_{2}\approx 0$$
  (c) Real MRI Slice
    For a real MRI slice, the contrast, sharpness and total quality scores in Figure 2h are defined to lie in the range of values between ideal and extremely degraded MRI slices:
    (17) $$\begin{array}{c} \left\{Q_{1}:0\leq Q_{1}\leq 1\right\}\\ \left\{Q_{2}:0\leq Q_{2}\leq 1\right\}\\ \left\{Q_{T}:0\leq Q_{T}\leq 1\right\} \end{array}$$

## 3. Experiment

### 3.1. Sources and Description of Test Data

The dataset for the experiment consist of 18 brain, ten cardiac and ten breast MRI volume data. The brain data consist of 15 real and three simulated datasets. In all the MRI data file information, spatial resolution was not explicitly expressed by the field of view (FOV). The available metric closest to the FOV is slice thickness.

#### 3.1.1. Real Brain MRI Data

The real brain MRI volume data were acquired without perceived distortion. It was provided by NeuroRx Research Inc. (https://www.neurorx.com), BrainCare Oy (http://braincare.fi/) and The Alzheimer’s Disease Neuroimaging Initiative ADNI (www.adni.loni.usc.edu). They consist of 10 T2 images and 5 T1 images. Each slice in the T2 and T1 MRI volume data from NeuroRx has dimension of 256×256 and 2.4 mm thickness. Data from ADNI has dimension 192×166 and 1.2 mm thickness. The T1 MRI volume data from BrainCare has dimension 448×512 pixels and 5 mm thickness.

#### 3.1.2. Cardiac MRI Data

The cardiac MRI were short axis MRI volume data from the Department of Diagnostic Imaging of the Hospital for Sick Children in Toronto, Canada (http://www.sickkids.ca/DiagnosticImaging/index.html). The images were acquired using the Fast Imaging Employing Steady State Acquisition (FIESTA) sequence protocol. The data were among the experimental data in the report [32]. The report describes the framework for the analysis of short axis cardiac MRI using statistical models of shape and appearance. Each volume data has four dimensions and contains 20 frames. The number of slices in each frame varies from 8 to 15. The dimension of each slice is 256×256 along the long axis.

#### 3.1.3. Breast MRI Data

The breast MRI was obtained from the Reference Image Database to Evaluate Therapy Response (RIDER) [33] in the Cancer Imaging Archive (TCIA) (https://wiki.cancerimagingarchive.net/display/Public/RIDER+Breast+MRI#) database. Each volume data contains 60 slices with dimension 288×288 and thickness of 2.5 mm.

#### 3.1.4. Simulated MRI Data

Three simulated T1, T2 and PD MRI volume data were downloaded from Mcgill University BrainWeb [34]. There are 60 slices in each volume data. Each slice has dimension 217×181 and thickness of 3 mm.

### 3.2. Generation of Noise Distortion

Noise was added to a real MRI image according to the procedure outlined in [5]. Two separate and identical realizations of white Gaussian noise N(0,σ) were generated. The standard deviation σ of the Gaussian noise is expressed as a percentage τ of the maximum intensity level ν in the image. Thereafter, we simulate the complex plane of MRI acquisition process. The real component $I_{r}$ was simulated by adding a realization of the Gaussian noise to a real MRI image *A*:

(18) $$I_{r}=A+N\left(0,\sigma \right)$$

The imaginary component $I_{i}$ is the second realization of the Gaussian noise:

(19) $$I_{i}=N\left(0,\sigma \right)$$

Added Rician noise *m* is the magnitude of the complex data.:

(20) $$m=\sqrt{I_{r}^{2}+I_{i}^{2}}$$

White Gaussian noise equivalent of Rician noise level is defined as in [5]:

(21) $$\sigma =\frac{\nu \tau }{100}$$

The Rician noise level was scaled from 1 to 15 in unit step.

### 3.3. Experiment Category

Objective and subjective evaluation are the two main categories of the experiment.

#### 3.3.1. Objective Evaluation

The objective evaluation has three categories; retrospection, noise reduction and comparative performance evaluation.

1. Retrospection
  This category utilize real MRI data that were retrospectively acquired without degradation. It was further divided into T2, T1, breast and cardiac MRI data. 250 slices were utilized for each category.
2. Noise Reduction
  Two hundred slices were selected from the retrospectively acquired MRI data for performance evaluation of the bilateral filter proposed in [35]. The bilateral filter is a non-linear filter which became popular because of its edge-preserving feature. We chose to evaluate only one state-of-the-art noise reduction algorithm because the goal of this research is not comparative performance evaluation. The MATLAB implementation code was downloaded from (people.csail.mit.edu/jiawen/software/). Our proposed method assessed the noise reduction algorithm at mild (4 percent), moderate (8 percent) and severe (12 percent) levels of Rician noise. The parameters of the bilateral filter are as follows; smoothing parameter in the spatial dimension $\sigma_{S}=5$, smoothing parameter in the range dimension $\sigma_{R}=15$, amount of downsampling in the spatial dimension $\gamma_{R}=5$ and amount of downsampling in the range dimension $\gamma_{S}=15$.
3. Comparative Performance Evaluation
  Comparative performance evaluation was carried out on simulated and real MRI data. The simulated data were 15 slices from T1, T2 and PD MRI volume data. The real data were retrospectively acquired from 15 T2, 15 T1, 15 breast and ten cardiac MRI images. The proposed method was compared to SSIM, PSNR and BRISQUE. Rician noise, from level 0 to level 15 was added to each slice in a MRI volume. For each level of noise, quality prediction from each quality metric is the average quality scores from all slices in the MRI volume.
  The SSIM and our proposed method have the same lower and upper limit quality indices. This is not the case for PSNR and BRISQUE. The quality indices from the PSNR and BRISQUE were modified to have same lower and upper limit quality indices as our proposed method. Since PSNR will give very large number, it was computed in the decibel scale. The decibel value was further divided by 100. BRISQUE quality index is such that the image with best quality is 0 while that image with worst quality is 100. To make the quality index comparable to our proposed method, output from BRISQUE was subtracted from 100. The difference is further divided by 100.

#### 3.3.2. Subjective Validation

The objective experiment was validated by four observers; two radiologists and two medical imaging professionals. The validation experiment was facilitated by **QuickEval** [36], a web-based tool for psychometric image evaluation provided by the Norwegian Colour and Visual Computing Laboratory (www.colourlab.no/quickeval) at the Norwegian University of Science and Technology, Gjovik, Norway. The experiment was classified according to the objective experiment category, but we report results for only comparative performance evaluation.

The observer assigns a score between 0 and 100, in unit step, to each slice. Each score assigned by the observer is divided by 100 to ensure that the subjective and objective scales are in the same range. An observer was first presented with an undistorted version of an MRI slice, followed by increasing distortion levels in the original slice. The distorted levels are 5, 10 and 15. Spearman’s rank correlation coefficient ρ [37] is the metric we use to measure the relationship between our objective results and the score assigned by human observers.

## 4. Results

Noise, like other classical quality attributes contributes to the visual quality of an image. This research exploit the principle of local indicators of spatial autocorrelation for blind assessment of the perceptual quality of magnitude MRI images in environment where Rician noise is the dominant type of distortion. Contrast and sharpness quality of an image is expressed by the degree of clustering within the image. The degree of clustering is quantitatively expressed by local Morgan statistics.

The proposed noise-based quality evaluation method was implemented in the MATLAB computing environment. The demonstration and implementation codes are attached as supplementary file to this paper. Upon acceptance, the codes will be available for download from (www.colourlab.no/software). In this paper, nine figures are used to explain the performance evaluation of the proposed method.

**Figure 3:**
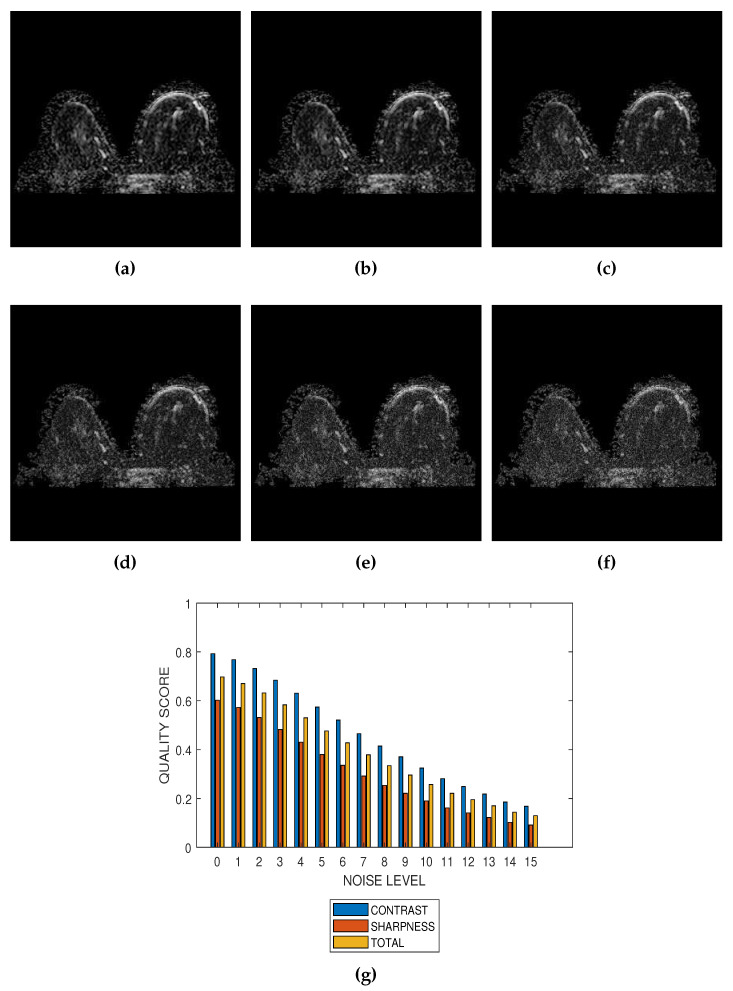
(**a**) A breast MRI slice provided by the RIDER project of The Cancer Imaging Archive (TCIA) and its degraded versions at Rician noise levels (**b**) 3 percent, (**c**) 6 percent, (**d**) 9 percent, (**e**) 12 percent and (**f**) 15 percent. (**g**) Variation of contrast, sharpness and total quality scores for Rician noise levels that vary from 0 percent to 15 percent.

**Figure 4:**
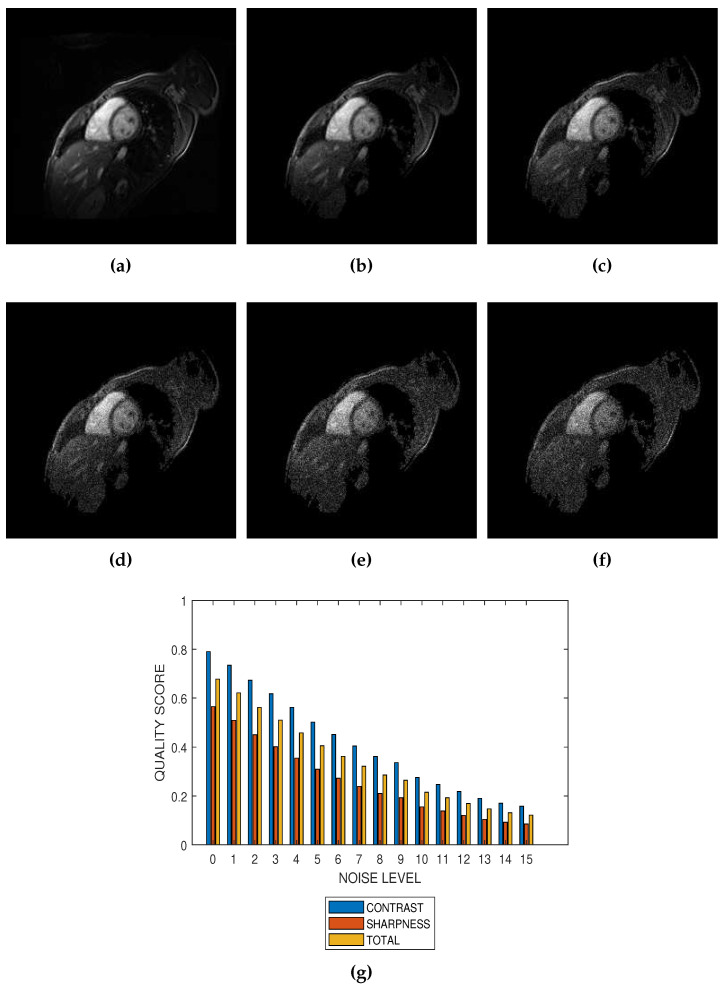
(**a**) A short axis cardiac MRI slice provided by the Department of Diagnostic Imaging of the Hospital for Sick Children in Toronto, Canada, and its degraded versions at Rician noise levels (**b**) 3 percent, (**c**) 6 percent, (**d**) 9 percent, (**e**) 12 percent and (**f**) 15 percent. (**g**) Variation of contrast, sharpness and total quality scores for Rician noise levels that vary from 0 percent to 15 percent.

**Figure 5:**
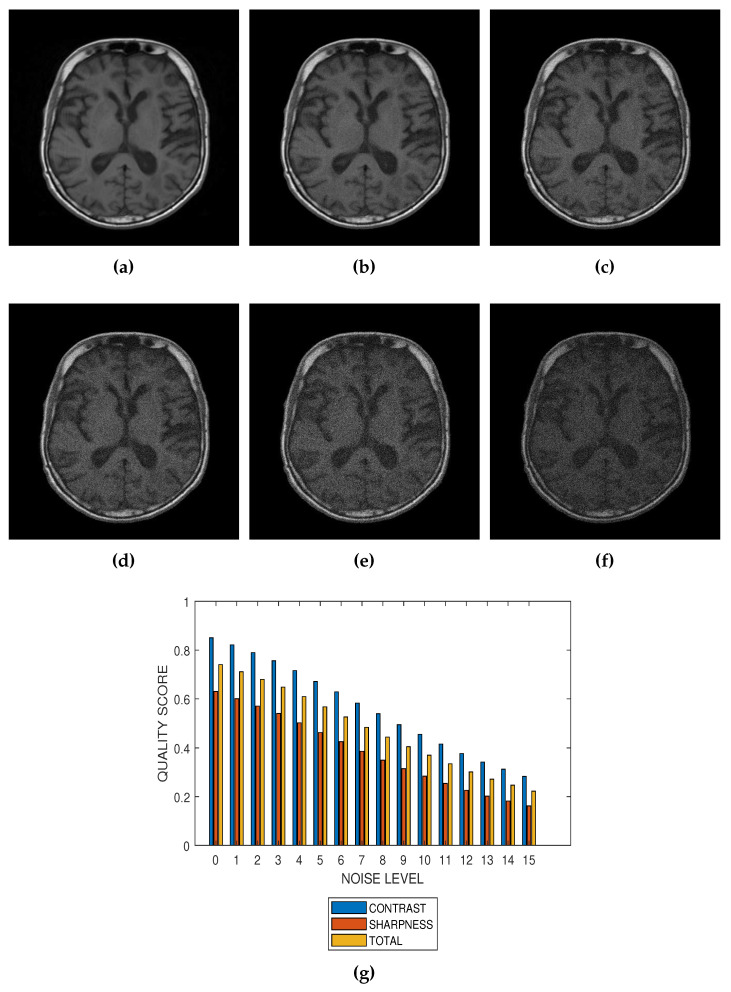
(**a**) A conventional T1 weighted MRI slice from BrainCare Oy., and its degraded versions at Rician noise levels (**b**) 3 percent, (**c**) 6 percent, (**d**) 9 percent, (**e**) 12 percent and (**f**) 15 percent. (**g**) Variation of contrast, sharpness and total quality scores for Rician noise levels that vary from 0 percent to 15 percent.

**Figure 6:**
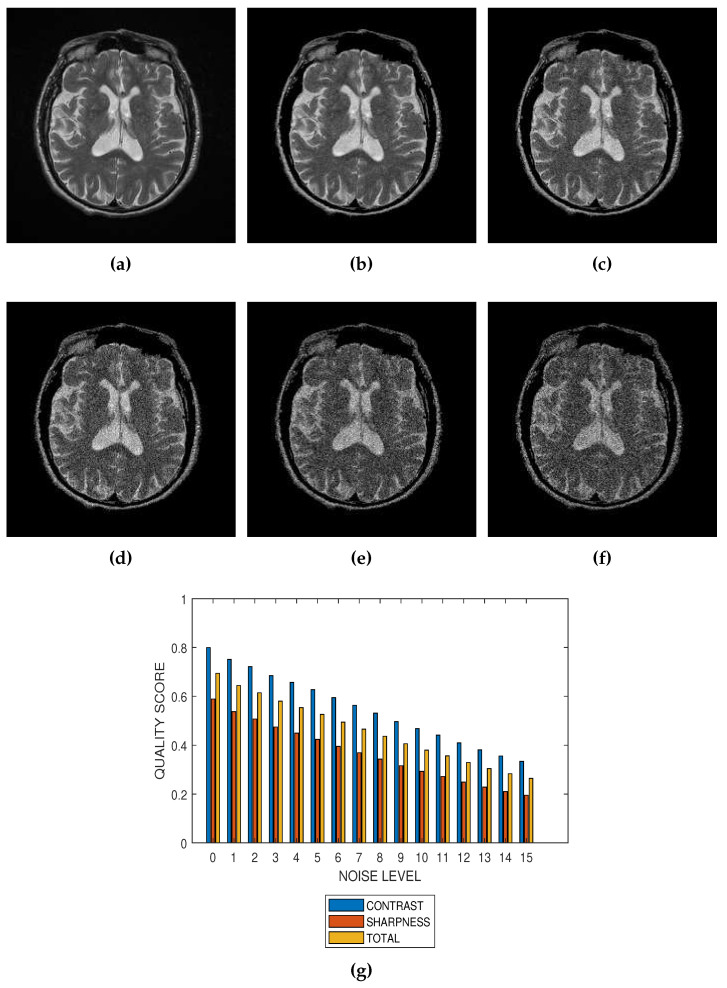
(**a**) A T2 weighted MRI slice from ADNI and its degraded versions at Rician noise levels (**b**) 3 percent, (**c**) 6 percent, (**d**) 9 percent, (**e**) 12 percent and (**f**) 15 percent. (**g**) Variation of contrast, sharpness and total quality scores for Rician noise levels that vary from 0 percent to 15 percent.

The first slice images (Figure 3a, Figure 4a, Figure 5a and Figure 6a) in Figure 3, Figure 4, Figure 5 and Figure 6 are the original images without degradation. The following five slice images are Rician noise degraded versions of the original images at Rician noise levels of 3 percent, 6 percent, 9 percent, 12 percent and 15 percent, respectively. For each level of noise, the contrast, sharpness and total quality scores are displayed in Figure 3g, Figure 4g, Figure 5g and Figure 6g. Expectedly, the quality scores decrease with increasing level of noise.

**Figure 7:**
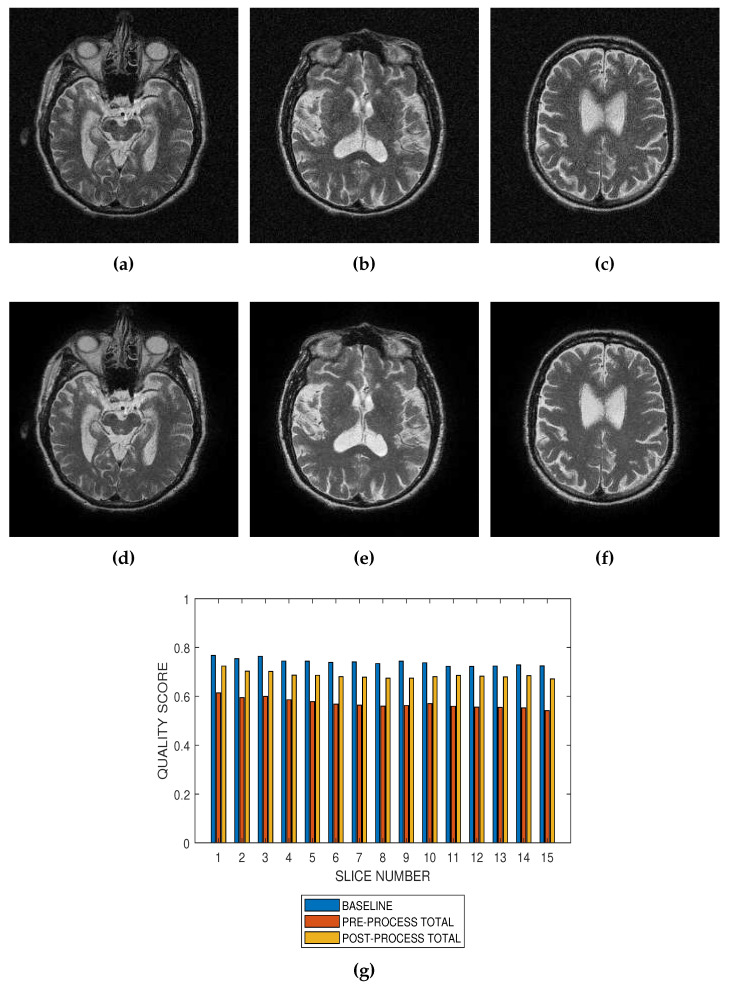
Application of the proposed method to the assessment of an image enhancement algorithm. (**a**–**c**) are slices numbers 20, 27 and 34, respectively in a T2 MRI volume degraded with 4 percent Rician noise. (**d**–**f**) are the slices displayed in (**a**–**c**) but their quality have been enhanced using a bilateral filter. (**g**) The baseline quality score of the original image, the predicted total quality indices of 12 successive T2 MRI slices in a volume data before and after enhancement with a bilateral filter.

**Figure 8:**
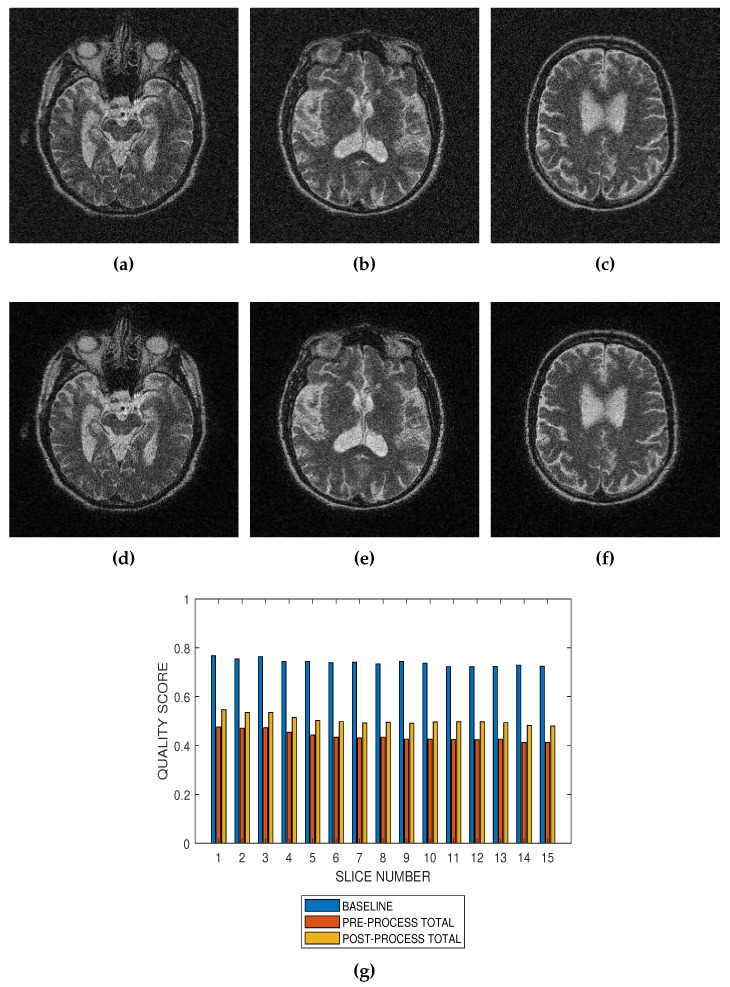
Application of the proposed method to the assessment of an image enhancement algorithm. (**a**–**c**) are slices numbers 20, 27 and 34, respectively in a T2 MRI volume degraded with 8 percent Rician noise. (**d**–**f**) are the slices displayed in (**a**–**c**) but their quality have been enhanced using a bilateral filter. (**g**) The baseline quality score of the original image, the predicted total quality indices of 12 successive T2 MRI slices in a volume data before and after enhancement with a bilateral filter.

**Figure 9:**
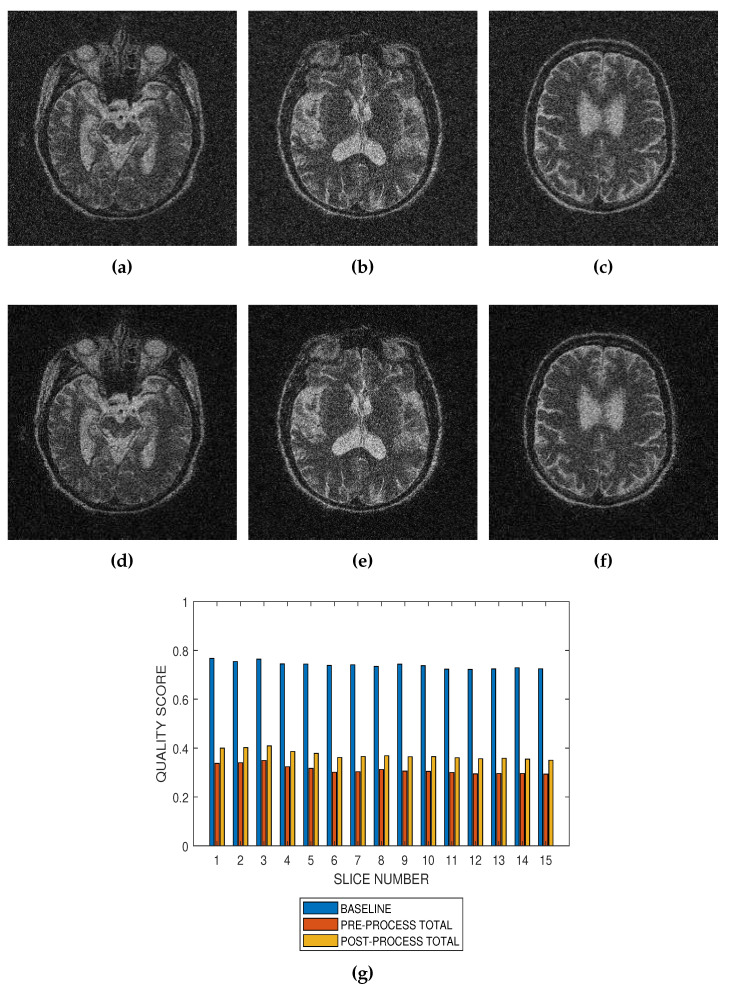
Application of the proposed method to the assessment of an image enhancement algorithm. (**a**–**c**) are slices numbers 20, 27 and 34, respectively in a T2 MRI volume degraded with 12 percent Rician noise. (**d**–**f**) are the slices displayed in (**a**–**c**) but their quality have been enhanced using a bilateral filter. (**g**) The baseline quality score of the original image, the predicted total quality indices of 12 successive T2 MRI slices in a volume data before and after enhancement with a bilateral filter.

Performance of the noise reduction algorithm at 4 percent, 8 percent and 12 percent noise levels are displayed in Figure 7, Figure 8 and Figure 9, respectively. In the three levels of Rician noise, the post processing quality scores are higher than corresponding preprocessing quality scores.

**Figure 10:**
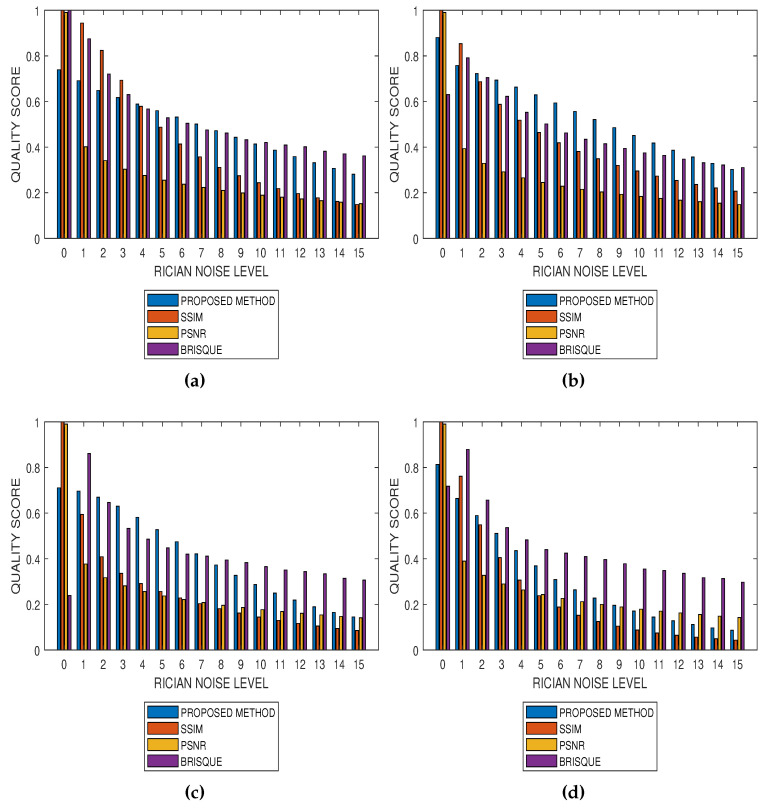
Comparative performance evaluation of the proposed method with SSIM, PSNR and BRISQUE quality metrics using (**a**) 15 slices in a T2 MRI volume data, (**b**) 15 slices in a T1 MRI volume data, (**c**) 15 slices in a breast MRI volume data and (**d**) 10 slices in a short axis cardiac MRI volume data.

**Figure 11:**
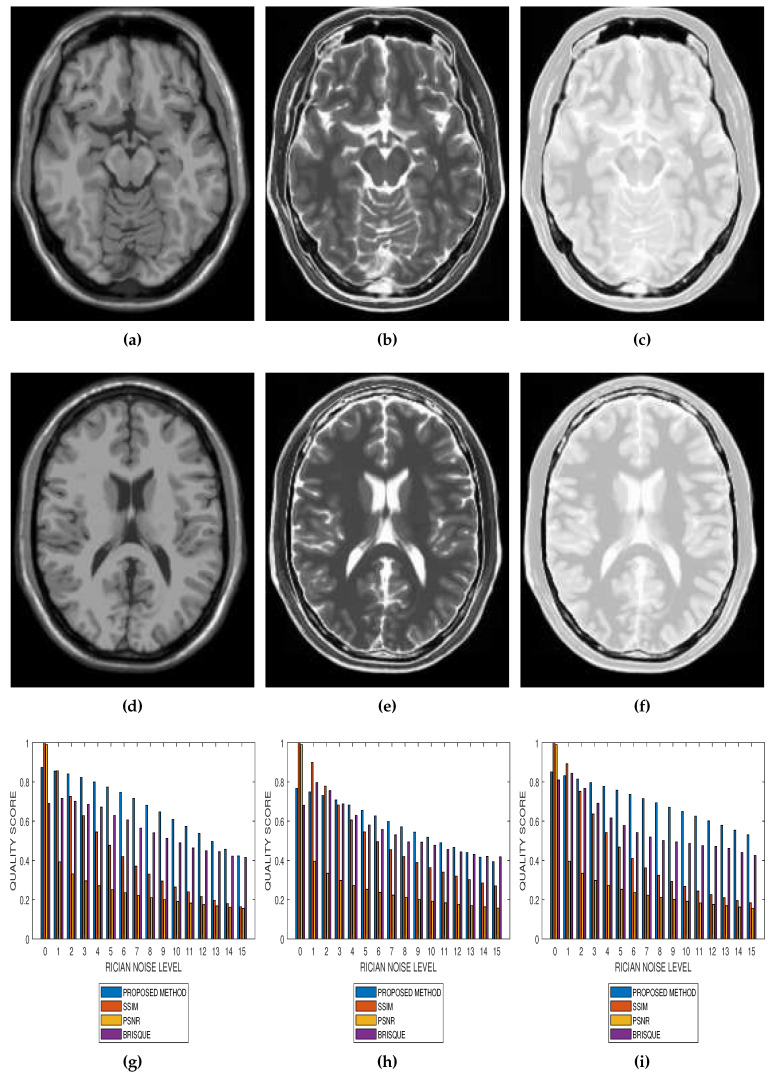
Comparative performance evaluation of the proposed method with SSIM, PSNR and BRISQUE quality metrics using 15 slices in (**a**,**d**) T1, (**b**,**e**) T2 and (**c**,**f**) PD simulated MRI volume data from McGill University BrainWeb (**g**–**i**).

Results from comparative performance evaluation of the proposed method with SSIM, PSNR and BRISQUE are displayed in Figure 10 and Figure 11, for real and simulated MRI data, respectively. Figure 10a–d are quality predictions on T2, T1, breast and cardiac MRI slices for Rician noise levels from 0 to 15. The images in the first, second and third columns of Figure 11 are sample MRI images from simulated T1, T2 and PD MRI volume data. Quality predictions corresponding to each MRI volume data, for Rician noise levels from 0 to 15, are the plots in the last row of Figure 11. For all the quality metrics, there is a general trend of decreasing quality score with increasing noise level. Table 1, Table 2, Table 3 and Table 4 are the subjective evaluation results from the comparative performance of our proposed method with SSIM, PSNR and BRISQUE quality metrics.

## 5. Discussion

The goal of image quality evaluation systems is to predict the quality of images with the same level of efficiency as human visual system (HVS). In this research, we propose to evaluate the quality of MRI images in a noise-dominant environment. The formulation of the proposed method incorporates four steps which mimic HVS characteristics. First, the magnitude of local Moran statistics at each location can be regarded as a measure of contrast sensitivity threshold. Second, the spatial weight which define the spatial interaction in a local neighbourhood accounts for the visibility of locally perceived noise. Third, averaging the local Moran statistics to give the global Moran statistics is the equivalent of integrating perceptually weighted local noise. Fourth, perceptual weight, expressed as a function of GMS is assigned to contrast and sharpness quality attributes in the computation of the total quality score. The proposed quality evaluation system transforms noise level into an easy-to-interpret and standardized quality metric. The lower and upper limits of the quality index are 0 and 1, respectively. Below, we discuss the suitability of our proposed method to different applications.

The proposed method is a practical and realistic approach to quality evaluation. The images in Figure 3, Figure 4, Figure 5 and Figure 6 were evaluated without the need of a reference image. The images used for the performance evaluation were acquired with different levels of details and, hence, possess different perceptual quality. Variation in the profiles of quality scores shown in Figure 3, Figure 4, Figure 5 and Figure 6 shows that the proposed quality evaluation system can provide fairly good perceptual quality assessment for different types of MRI images.

Performance evaluation of noise reduction algorithms will be a useful application of the proposed method. Evaluation of the proposed methods before and after denoising MRI images (See Figure 7, Figure 8 and Figure 9) show that the performance of noise reduction algorithms decreases with the severity of noise.

There is no doubt that SSIM, PSNR and BRISQUE are popular and efficient quality evaluation techniques. The comparative performance evaluation results displayed in Table 1, Table 2, Table 3 and Table 4 as well as Figure 10 and Figure 11 reveal their limitations and add to the growing calls for application-specific quality metrics. At lower noise levels, current techniques provide an inaccurate estimate of perceptual quality. This is expected for SSIM and PSNR as their predicted quality score is based on noise level and with reference to an assumed ideal image. The no-reference BRISQUE performed better than SSIM and PSNR. However, inaccurate estimation of noise, particularly at lower noise levels in T2, T1 and Breast MRI, was evident in BRISQUE. This can be attributed to training data extracted from images corrupted by Gaussian noise rather than the Rician noise present in MRI images. The proposed method has the lowest margin between a predicted quality score and the corresponding score assigned by human observers. Furthermore, it has the highest perceptual quality discrimination for different noise levels. For these reasons, we can say that the proposed method demonstrates superior performance over current techniques.

A good image quality evaluation model should not only deliver high quality prediction accuracy but also be computationally efficient [38]. The proposed method meets these requirements. Unlike some current techniques such as BRISQUE, there is no need for additional resources such as complex feature extraction and training of data for quality prediction. The simple and efficient feature extraction coupled with processing in the binary domain makes the proposed method useful in applications where large volumes of MRI data are processed.

Evaluation of the proposed method on real MRI images was complemented with anatomically realistic phantoms. Since spatially invariant Rician noise was artificially added to these images, the performance of the proposed method on spatially variant noise in multiple-coil acquired MRI images, remains an open question. However, the performance evaluation results demonstrate the promise of a new state-of-the-art algorithm.

## 6. Conclusions

Noise limits the utility of medical images for the diagnosis of diseases. This paper propose a new noise-based quality evaluation for MRI images. The LMS estimates the visibility of locally perceived noise from the clustered and dispersed pixels. Noisiness at each local area is perceptually weighted using the spatial weight matrix. The perceptually weighted local noise is integrated to form the GMS. Perceptual weight assigned to the contrast and sharpness quality scores is expressed as a function of the GMS. Quality prediction is based on the perceptually weighted sum of the clustered and dispersed pixels in the image. The proposed method is computationally efficient and performance evaluation shows good correlation with subjective evaluation by human observers. Potential applications of the proposed method include the evaluation of retrospectively acquired MRI images, performance evaluation of noise reduction algorithms, parameter optimization for denoising algorithms and the selection of an appropriate acceleration factor in parallel imaging acquisition techniques.

## Figures and Tables

**Table 1:** Comparative Performance Evaluation of the Proposed Method with SSIM, PSNR and BRISQUE Quality Metrics using T2 MRI Sequence.

Noise Level	Number of Slices	Average Subjective Score	Average Objective Score			
			Proposed Method	SSIM	PSNR	BRISQUE
0	15	0.70	0.75	1.00	0.95	1.00
5	15	0.65	0.60	0.60	0.30	0.60
10	15	0.55	0.50	0.30	0.25	0.50
15	15	0.40	0.35	0.20	0.20	0.40

**Table 2:** Comparative Performance Evaluation of the Proposed Method with SSIM, PSNR and BRISQUE Quality Metrics using T1 MRI Sequence.

Noise Level	Number of Slices	Average Subjective Score	Average Objective Score			
			Proposed Method	SSIM	PSNR	BRISQUE
0	15	0.81	0.85	1.00	0.90	0.62
5	15	0.73	0.70	0.52	0.30	0.60
10	15	0.50	0.55	0.35	0.20	0.45
15	15	0.35	0.40	0.30	0.18	0.40

**Table 3:** Comparative Performance Evaluation of the Proposed Method with SSIM, PSNR and BRISQUE Quality Metrics using Breast MRI Images.

Noise Level	Number of Slices	Average Subjective Score	Average Objective Score			
			Proposed Method	SSIM	PSNR	BRISQUE
0	15	0.75	0.70	1.00	0.95	0.23
5	15	0.61	0.55	0.30	0.25	0.50
10	15	0.40	0.35	0.20	0.22	0.40
15	15	0.20	0.15	0.15	0.15	0.35

**Table 4:** Comparative Performance Evaluation of the Proposed Method with SSIM, PSNR and BRISQUE Quality Metrics using Cardiac MRI Images.

Noise Level	Number of Slices	Average Subjective Score	Average Objective Score			
			Proposed Method	SSIM	PSNR	BRISQUE
0	10	0.76	0.80	1.00	0.95	0.70
5	10	0.47	0.45	0.32	0.27	0.50
10	10	0.25	0.20	0.10	0.10	0.40
15	10	0.15	0.10	0.05	0.03	0.35

## Abbreviations

MRI	Magnetic Resonance Imaging
SNR	Signal-to-Noise Ratio
PSNR	Peak Signal-to-Noise Ratio
MSE	Mean Square Error
RMSE	Root Mean Square Error
ADNI	The Alzheimer’s Disease Neuroimaging Initiative
RIDER	Reference Image Database to Evaluate Therapy Response
TCIA	The Cancer Imaging Archive
HVS	Human Visual System
LMS	Local Moran Statistics
GMS	Global Moran Statistics
P1	Parallel Imaging
T1	Longitudinal Relaxation
T2	Transverse Relaxation
